# Sociodemographic Characteristics Associated with Harmful Use of Alcohol Among Economically and Socially Disadvantaged Immigrant Patients in Italy

**DOI:** 10.1007/s10903-019-00928-z

**Published:** 2019-08-09

**Authors:** Anteo Di Napoli, Teresa Morgillo, Alessandra Rossi, Martina Ventura, Lorenzo Nosotti, Andrea Cavani, Gianfranco Costanzo, Concetta Mirisola, Alessio Petrelli

**Affiliations:** National Institute for Health, Migration and Poverty (INMP), Via di San Gallicano, 25/a, 00153 Rome, Italy

**Keywords:** Alcohol, Socioeconomic, Immigrants, Mental disorders, Italy

## Abstract

In many contexts, individuals with lower socioeconomic status, especially immigrants, have a higher burden of negative alcohol-related consequences and a higher probability of receiving a psychiatric diagnosis. This study aimed at exploring sociodemographic and clinical characteristics associated with harmful use of alcohol (HUA) among immigrant patients. A cross-sectional study was conducted in Rome (Italy) on a sample of 330 immigrant patients admitted to the gastroenterology outpatient clinic of the INMP (March 2013–October 2014). HUA was evaluated through the Alcohol Use Disorders Identification Test (AUDIT) questionnaire. The presence of psychiatric disorders was diagnosed through SCID I–II interviews. The association between sociodemographic characteristics and psychiatric disorders and HUA was evaluated through a multivariate log-binomial regression model. HUA was associated with unemployment, longer stay in Italy, mood disorder and not being married, especially among African immigrants. We provide original findings about a selected, hard-to-investigate population, suggesting priorities in interventions on HUA among specific vulnerable subgroups.

## Introduction

The harmful use of alcohol (HUA) is one of the leading risk factors for population health; it is associated with a high risk of health problems with acute and long-term effects and accounts for 3 million deaths worldwide every year (3% of premature deaths) [1, 2].

A country’s prevalence of alcohol consumption is associated with demographic and socioeconomic factors, level of economic development, religion and cultural norms and the types of alcoholic beverage commonly consumed [1, 3]. However, the relationship between socioeconomic status (SES) and alcohol consumption is complex, due among other things to the wide variety of definitions of drinking and of SES [1, 3]. Previous studies found that people with higher SES may consume similar or greater amounts of alcohol compared with people with lower SES, but the latter group has a higher burden of negative alcohol-related consequences and a higher probability of receiving a psychiatric diagnosis [1–3].

Moreover, the association between SES and alcohol use and health-related outcomes is further complicated by a variety of moderating factors, such as sex, race and ethnicity [2]. In particular, immigrants may experience more vulnerability to HUA due to critical issues of the integration process in the host country. Research has shown that acculturation, defined as ‘the process whereby immigrants change their behavior and attitudes towards those of the host society’, may play an important role in the alcohol consumption behaviors of certain racial/ethnic groups [4].

In Italy, 14.8% of men and 6.2% of women aged 11 years or more habitually exceed the maximum daily intake of alcohol recommend by the Ministry of Health [5]. Among foreign citizens aged 14 years or more, 13.2% have presented unhealthy drinking behaviours, with an incidence three time higher in men (20.1%) than in women (7.1%). A higher prevalence of hazardous drinking has been observed among immigrants from Eastern Europe than among those from Asia and Africa, who presented the lowest level of consumption [6].

However, few data are available about disadvantaged and vulnerable groups of the population (e.g. the poor, regular and undocumented immigrants, victims of violence and trade, international protection applicants), who often encounter difficulties accessing health and social care system.

The aim of the present study was to explore sociodemographic and clinical characteristics associated with HUA in a sample of vulnerable immigrant patients from different countries of origin within the context of a social medicine project conducted in Rome.

## Methods

### Study Design, Setting and Study Population

This cross-sectional study was conducted by the Italian National Institute for Health Migration, and Poverty (INMP), based in Rome, within the framework of a social medicine project funded by the Ministry of Health targeting economically and socially disadvantaged subjects with liver disease. INMP uses a holistic health and social care system and patterns of care with a transcultural approach to facilitate access to health care services.

The study was conducted on a sample of 330 consecutive immigrant patients enrolled in the social medicine project; they were aged at least 18 years and were admitted to INMP’s gastroenterology outpatient clinic between March 2013 and October 2014. Immigrant status was defined through citizenship.

Anamnestic evidence of HUA consumption was evaluated through the Alcohol Use Disorders Identification Test (AUDIT) questionnaire, a screening tool developed by the WHO to assess alcohol consumption, drinking behaviours and alcohol-related problems in a primary healthcare setting [7]. Patients were encouraged to answer ten questions, each scored from zero to four on a Likert scale, with a total score ranging from 0 to 40. A score ≥ 8 indicates HUA. Patients’ sociodemographic information was collected during the clinical examination.

The presence of psychiatric disorders was diagnosed through semi-structured SCID I and II interviews from DSM-IV [8].

### Statistical Analysis

We described sociodemographic characteristics and psychiatric disorders of the study population. The differences between subjects from the two most frequent areas of origin (Africa and Eastern Europe) were evaluated. The association between sociodemographic characteristics and psychiatric disorders with HUA (presence/absence) was analysed through a multivariate log-binomial regression model. We also tested the interaction between marital status and area of origin. All the variables considered for the analysis are shown in the tables.

### Ethics

The project was reviewed and approved by the Italian Ministry of Health. The study design was retrospective and not experimental, and information was routinely collected. All patients signed an informed consent and INMP Review Board approved the study.

## Results

Table 1 summarizes the characteristics of the study population. The 330 patients included in the study were mainly men (86.7%) and had a mean age of 42.2 ± 10.8 (standard deviation) years. Concerning the country of origin, most of the patients came from Eastern Europe and Africa (48.2% and 34.5%, respectively), whilst only 9.7% came from Asia and 7.6% from the Americas. The median permanence in Italy was 7 years, 61.5% were unemployed, 59.4% had a low education level and only 31% were married. The presence of mood disorders or other psychopathological disorders was diagnosed in about one-third of the study population.

**Table 1:** Sociodemographic and clinical characteristics of the study population

	n	%
Sex		
Men	286	86.7
Women	44	13.3
Age (years)		
18–34	85	25.8
35–54	204	61.8
≥ 55	41	12.4
Length of stay (years)		
< 2	45	13.6
2–5	98	29.7
6–10	94	28.5
> 10	93	28.2
Area of origin		
Africa	114	34.5
The Americas	25	7.6
Asia	32	9.7
Eastern Europe	159	48.2
Occupational status		
Employed/retired	127	38.5
Unemployed	203	61.5
Education level^a^		
Medium/high	134	40.6
Low	196	59.4
Marital status		
Married	102	30.9
Not married	228	69.1
Mood disorders		
Absent	222	67.3
Present	108	32.7
Other psychopathological disorders		
Absent	217	65.8
Present	113	34.2

^a^We classified education in three levels based on number of years of schooling: low (up to 8 years), medium (up to 13 years) and high (more than 13 years)

When focusing on the characteristics of patients from the two most frequent areas of origin—Africa and Eastern Europe—some differences were found (Table 2): immigrants from Eastern Europe were more frequently employed or retired than were Africans (44.7% vs 28.9%, p < 0.01) and they had more often a “medium or high” education level (52.2% vs 28.1, p < 0.0001). Furthermore (data not shown), the African patients compared with those from Eastern Europe were younger (mean ± SD: 36.1 ± 9.4 vs 47.1 ± 9.6, p < 0.0001) and had a shorter length of stay in Italy (median: 3 vs 9 years, p < 0.0001).

**Table 2:** Sociodemographic and clinical characteristics of the study population from Africa and Eastern Europe

	Africa (n = 114)		Eastern Europe (n = 159)		Total (n = 330)		p value
	n	%	n	%	n	%	
Sex							
Men	104	91.2	127	79.9	231	84.6	0.010
Women	10	8.8	32	20.1	42	15.4	
Age (years)							
18–34	54	47.4	14	8.8	68	24.9	< 0.0001
35–54	54	47.4	114	71.7	168	61.5	
≥ 55	6	5.3	31	19.5	37	12.1	
Length of stay (years)							
< 2	24	21.1	13	8.2	37	13.6	< 0.0001
2–5	49	43.0	30	18.9	79	28.9	
6–10	23	20.2	58	36.5	81	29.7	
> 10	18	15.8	58	36.5	76	27.8	
Occupational status							
Employed/retired	33	28.9	71	44.7	104	38.1	0.008
Unemployed	81	71.1	88	55.3	169	61.9	
Education level							
Medium/high	32	28.1	83	52.2	115	42.1	< 0.0001
Low	82	71.9	76	47.8	158	57.9	
Marital status							
Married	38	33.3	44	27.7	82	30.0	0.314
Not married	76	66.7	115	72.3	191	70.0	
Mood disorders							
Absent	82	71.9	99	62.3	181	66.3	0.096
Present	32	28.1	60	37.7	92	33.7	
Other psychopathological disorders							
Absent	73	64.0	113	71.1	186	68.1	0.219
Present	41	36.0	46	28.9	87	31.9	

Table 3 presents the crude and the adjusted prevalence ratios (PR) of HUA for the sociodemographic and clinical factors of the 330 patients. A higher adjusted prevalence of outcome was observed among unemployed individuals (PR = 1.49, p = 0.02), among those who had been in Italy longer than 7 years (PR = 1.50, p = 0.01) and among patients with a mood disorder (PR = 1.46, p = 0.03). We found a lower risk of HUA in patients with other psychopathological disorders at univariate analysis (PR = 0.66; p = 0.02), although this association was not confirmed after adjusting for the other factors (PR = 0.83, p = 0.38).

**Table 3:** Factors associated with harmful use of alcohol (HUA)

	HUA (%)	Crude PR	95%CI	Adjusted PR	95%CI	*p* value
Sex						
Men	36.7	1.00	–	1	–	
Women	36.4	0.99	(0.65–1.51)	1.07	(0.69–1.64)	0.768
Age (years)						
18–34	28.2	1.00	–	1.00	–	
35–54	38.2	1.35	(0.93–1.98)	1.25	(0.80–1.94)	0.321
≥ 55	46.3	1.64	(1.02–2.63)	1.16	(0.68–1.98)	0.581
Area of origin						
Africa	32.5	1.00	–	1	–	
The Americas	36.0	1.11	(0.62–1.99)	1.13	(0.66–1.94)	0.657
Asia	31.3	0.96	(0.41–1.72)	1.12	(0.63–1.98)	0.709
Eastern Europe	40.9	1.26	(0.91–1.74)	0.93	(0.69–1.25)	0.622
Length of stay (years)						
≤ 7	27.9	1.00	–	1	–	
> 7	47.0	1.68	(1.26–2.25)	1.50	(1.09–2.08)	0.013
Occupational status						
Employed/retired	27.6	1.00	–	1	–	
Unemployed	42.4	1.54	(1.11–2.13)	1.49	(1.06–2.09)	0.023
Education level						
Medium/high	39.6	1.00	–	1	–	
Low	34.7	0.88	(0.66–1.17)	0.99	(0.76–1.3)	0.939
Marital status						
Married	24.5	1.00	–	1	–	
Not married	42.1	1.72	(1.18–2.49)	1.64	(1.13–2.39)	0.010
Mood disorders						
Absent	28.8	1.00	–	1	–	
Present	52.8	1.83	(1.39–2.41)	1.46	(1.04–2.06)	0.030
Other psychopathological disorders						
Absent	41.5	1.00	–	1	–	
Present	27.4	0.66	(0.47–0.93)	0.83	(0.55–1.26)	0.380

Crude and adjusted prevalence ratios (PR) with 95%CI. Results from a multivariate log-binomial model

Not being married (PR = 1.64, p = 0.01) was also associated with an increased risk of HUA, especially among immigrants from Africa (PR: 2.38, p = 0.02) (data not shown).

## Discussion and Conclusions

The present study provides original findings on sociodemographic and clinical factors associated with HUA within a subgroup of hard-to-investigate immigrants who often encounter difficulties accessing health and social care system. The INMP healthcare model makes it possible to reach this population as it ensures free access to health and social services.

In particular, we found that in a sample of economically and socially disadvantaged immigrants in Italy, HUA was independently associated with not being married, being in an unstable employment situation and having a length of stay in Italy of at least 7 years. All these factors seem to be related to a failure of the immigration process, intended as the life projects and expectations of those individuals who decide to change their life. This failure may be particularly burdensome for those living for extended periods of time in the new host country, length of stay being considered an acculturation proxy variable as it is associated with the increased chance of deprivation condition, which is a risk factor for unhealthy lifestyles and worse health status [4]. Moreover, immigrant integration projects are usually work-oriented, devised and implemented to guarantee economic survival, obtain personal and professional satisfaction, obtain rights connected to the residence visa and improve social inclusion by becoming part of the host country. We also confirmed the evidence demonstrating an association between unemployment and abuse of alcohol, probably due to the social vulnerability generated by the lack of work or precarious employment [2].

The importance of a social network seems to be confirmed by the finding of a higher risk of HUA among the unmarried subjects, a condition that probably describes living alone. The impact of not being married is stronger among Africans than among Eastern European patients. The latter group is culturally closer to Italy and has a longer average stay, both factors being associated with a greater probability of social inclusion.

Furthermore, the proportion of men was higher among Africans than Eastern European patients. According to the marital resource model, marriage provides more benefits to men in the form of a healthy lifestyle, emotional support and physical comfort [9].

Finally, the finding of a higher risk of HUA among patients with mood disorders is consistent with other studies [10].

As our results are based on self-reported information, a limit of the study is that we could not rule out the possibility of under-reporting or over-reporting of alcohol consumption. Due to the cross-sectional design, a reverse causality in the association with HUA could be present for some factors, especially mental disorders, marital status and unemployment. Furthermore, a collider bias could be present because we selected patients who accessed a gastroenterology clinic, a condition potentially associated with both outcome and some risk factors.

Despite these limitations, this study contributes to the limited knowledge about social factors associated with alcohol abuse in a group of economically and socially disadvantaged immigrants who encounter barriers in accessing health and social care system.
